# Gastrojejunal Anastomosis Perforation after Gastric Bypass on a Patient with Underlying Pancreatic Cancer: A Case Report and Review of the Literature

**DOI:** 10.1155/2015/170901

**Published:** 2015-10-12

**Authors:** Omar Bellorin, Anna Kundel, Alexander Ramirez-Valderrama, Armando Castro

**Affiliations:** ^1^Department of General Surgery, New York Hospital Medical Center of Queens/Weill Cornell Medical College, 5645 Main Street, Flushing, NY 11355, USA; ^2^Department of Endocrine Surgery, New York University Langone Medical Center, 550 First Avenue, New York, NY 10016, USA

## Abstract

*Introduction*. We describe a case of gastrojejunal anastomosis perforation after gastric bypass on a patient with underlying pancreatic cancer. *Case Description*. A 54-year-old female with past surgical history of gastric bypass for morbid obesity and recent diagnosis of unresectable pancreatic cancer presents with abdominal pain, peritonitis, and sepsis. Computerized axial tomography scan shows large amount of intraperitoneal free air. The gastric remnant is markedly distended and a large pancreatic head mass is seen. Intraoperative findings were consistent with a perforated ulcer located at the gastrojejunal anastomosis and a distended gastric remnant caused by a pancreatic mass invading and obstructing the second portion of the duodenum. The gastrojejunal perforation was repaired using an omental patch. A gastrostomy for decompression of the remnant was also performed. The patient had a satisfactory postoperative period and was discharged on day 7. *Discussion*. Perforation of the gastrojejunal anastomosis after Roux-en-Y gastric bypass is an unusual complication. There is no correlation between the perforation and the presence of pancreatic cancer. They represent two different conditions that coexisted. The presence of a gastrojejunal perforation made the surgeon aware of the advanced stage of the pancreatic cancer.

## 1. Introduction

We present a case of a patient with history of pancreatic cancer and Roux-en-Y gastric bypass for morbid obesity who presented with acute abdomen secondary to perforation at the gastrojejunum anastomosis (GJA). The ideology, medical and surgical treatment, surveillance, and complications of GJA ulceration are reviewed. The relationship of obesity and cancer and its implications after gastric bypass is also addressed.

## 2. Case Description

**Figure 1:**
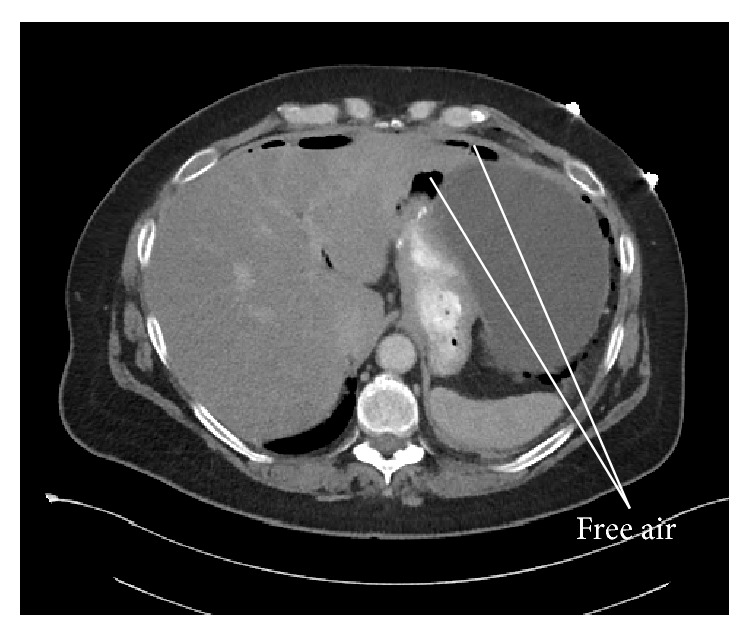
CT scan of abdomen showing moderate to large amount of intraperitoneal free air, likely representing bowel perforation.

**Figure 2:**
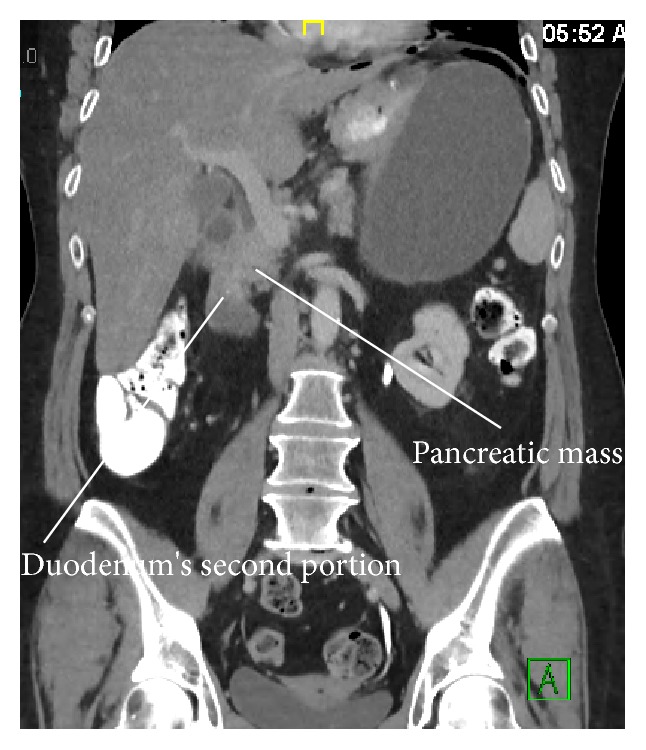
CT scan of abdomen demonstrating a pancreatic mass obstructing the second portion of the duodenum.

54-year-old female with past surgical history of antecolic antegastric Roux-en-Y gastric bypass in 2004 and recently diagnosed with unresectable pancreatic cancer status after chemotherapy presents with severe left upper quadrant abdominal pain and left sided chest pain which began 12 hours prior. Her current BMI is 35 and she still receives treatment for hypertension and diabetes. The pain was sudden in onset, sharp, and radiating to left shoulder. She had no previous episodes and had no recent esophagogastroduodenoscopy (EGD). Physical exam reveals tachycardia, tachypnea, hypotension, and left upper quadrant tenderness and guarding consistent with peritonitis. Computerized axial tomography (CAT) of the abdomen shows moderate to large amount of intraperitoneal free air, likely representing bowel perforation. The gastric remnant is markedly distended and the free air is only seen in the upper abdomen (Figure 1). There is a pancreatic mass obstructing the second portion of the duodenum (Figure 2).

The patient was resuscitated with intravenous fluid and given antibiotics and thereafter was brought to the operating room for exploratory laparotomy.

Intraoperative findings were consistent with a perforated ulcer located at the GJA and a distended gastric remnant caused by a pancreatic mass invading and obstructing the second portion of the duodenum. The abdominal cavity was thoroughly washed and the GJA perforation was repaired using an omental patch (Graham's). A gastrostomy for decompression of the remnant was also performed. The patient had a satisfactory postoperative period and was discharged on day 7.

## 3. Discussion

Patients with gastric bypass may experience a wide variety of complications that can be classified as acute or chronic. Marginal ulcers of the GJA usually represent a chronic complication that the bariatric surgeon encounters frequently.

The reported incidence of marginal ulceration of GJA after gastric bypass varies widely, ranging from 0 to 16% (Table 1). The risk factors for ulceration are smoking, use of nonsteroidal anti-inflammatory drugs (NSAIDs) and steroids, stress, recent surgery, and the presence of gastrogastric fistulas. Higher incidence has been reported in patients who underwent gastric bypass using circular staplers for the construction of the GJA as opposed to a linear stapler [1–4]. The use of nonabsorbable sutures in the GJA is also associated with marginal ulceration [5]. The presence of* Helicobacter pylori* may additionally play a role in the development of marginal ulcers. Schirmer et al. [6] described a 2.4% incidence of marginal ulcers in patients who underwent treatment for* Helicobacter pylori* preoperatively compared to those who did not (6.8%).

The majority of these ulcers can be treated medically. However, a subset of patients will have intractable disease requiring surgery for definitive management as the last resort. Patients with marginal ulcers are primarily medically treated with H2 blockers or proton pump inhibitors. Sucralfate is also added as well as smoking cessation and substitution of ulcerogenic medications. An upper gastrointestinal study is advised if a gastrogastric fistula is suspected and/or patients show no improvement after medical management. There is no consensus on the length of treatment, but the majority of bariatric surgeons choose a minimum of two to three months' regimen followed by an upper endoscopy for confirmation of resolution. Some surgeons advocate the endoscopic removal of nonabsorbable sutures, if present [5].

Clinically the symptoms suggestive of marginal ulceration include, but are not limited to, upper abdominal pain or progressive upper abdominal discomfort and intolerance of food and upper gastrointestinal bleed. Intractability is generally defined as persistence of symptoms after 3 months of medical treatment. Patel et al. [7] reported 39 patients with intractable marginal ulcers whose primary signs and symptoms included chronic abdominal pain (66.6%), GI bleeding (20.5%), stomal obstruction (10.2%), and perforation (2.5%). A minority of these patients will present with an acute abdomen similar to the patient we presented, and free perforation of the ulcer must be ruled out. Perforation of GJA ulcers is uncommon; the incidence ranges from 0.25 to 1% [7–10]. The risk factors for perforation are the same as for ulceration; however, smoking, history of recent surgery, and NSAIDs and steroids use are the common denominator in this particular situation [11]. They represent a life-threatening condition with a mortality rate of 10% [11].

Patients with perforation of a GJA ulcer need aggressive fluid resuscitation and prompt initiation of antibiotic therapy prior to any urgent surgical management. The definitive approach can be performed via open surgery or laparoscopy and consists of primary repair of the ulcer and omental patch along with a thorough washout of the abdominal cavity. A gastrostomy for feeding purposes should be performed as well. Large perforations not amenable to primary and patch repair may require revision of the gastrojejunal anastomosis.

The decision to use laparoscopic approach depended solely on the surgeon's expertise and confidence in advanced laparoscopy. There are several studies comparing open versus laparoscopic repair of perforated peptic ulcers that have demonstrated better outcomes in the laparoscopic group [16, 17]. Shorter hospital stay, reduced wound pain, and earlier return to normal activities are the main advantages. Kalaiselvan et al. [11] reported a series of 10 patients presented with perforated GJA ulcers. All patients were treated with abdominal washout, primary closure of the perforation, and omental patch. Five patients were operated on by general surgeons via an open approach and 5 underwent laparoscopic repair by bariatric surgeons. The laparoscopic group experienced lower morbidity, no mortality, and shorter hospital stay compared to those who underwent open surgery.

Postoperatively, these patients should have a reduction of risk factors, prolonged H2 blockers/proton pump inhibitors regimen, and eradication of* Helicobacter pylori* on those tested positive.

Also an upper endoscopy 3 months after the procedure to assess the GJA is recommended.

Obesity and cancer are strongly related. In the United States, approximately 85,000 new cases of patients with cancer per year are related to obesity [18]. Studies have found that an increase of body mass index by 5 kg/m^2^ is associated with a 10% higher cancer-related mortality. On the other hand, patients who undergo bariatric surgery have a lower incidence of cancer and a decrease in cancer-related mortality. This is presumably related to weight loss as demonstrated by Adams et al. [19] in a 12.5-year mean follow-up study of patients who underwent gastric bypass surgery compared to severely obese controls.

Early detection and treatment of cancer in patients undergoing bariatric surgery may also play a role. These patients undergo comprehensive gastrointestinal, pulmonary, and cardiovascular workup preoperatively that is not performed routinely in the general population. Moreover, the stomach remnant after gastric bypass will no longer be conveniently accessible, thus making the preoperative assessment of the upper gastrointestinal tract even more important. Zeni et al. [20] reported the presence of Barrett's esophagus in preoperative EGD to be 1.3%, GIST in 0.7%, gastric polyps in 5%, and* Helicobacter pylori*-associated gastritis and duodenitis in 27% and 6%, respectively. This extensive workup may result in early cancer diagnosis and is possibly part of the reason why patients undergoing obesity surgery have a lower cancer-related mortality.

Once a gastric bypass for obesity is performed, access to the gastric remnant and the biliary tree becomes complicated. There is no standard recommendation for a routine assessment of the gastric remnant after gastric bypass. Although technically difficult, double balloon enteroscopy is a feasible way to assess the duodenum and the residual stomach when the patient experiences symptoms that warrants further workup. Ultrasound or CT guided percutaneous gastrostomy and subsequent gastroscopy are another option. Combined laparoscopy-endoscopy can be used as last resort for diagnosis and treatment.

This anatomic exclusion certainly may result in a delayed diagnosis and treatment of a gastric/duodenal/pancreatic/periampular cancer. A locally advanced pancreatic mass in a patient who has undergone a gastric bypass may result in gastric outlet obstruction of the remaining stomach, which may lead to gastric distention, isquemia, and eventual perforation of the gastric remnant. A decompressive gastrostomy of the remnant is the treatment of choice to avoid this complication.

## 4. Conclusion

Perforation of the GJA after Roux-en-Y gastric bypass is an unusual complication. There is no correlation between the perforation and the presence of pancreatic cancer. They represent two different conditions that coexisted. The presence of a gastrojejunal perforation made the surgeon aware of the advanced stage of the pancreatic cancer that otherwise would have remained undetected for longer time. Overall the approach performed on the presented patient corresponds to the standard and the current literature. The laparoscopic approach by an experienced surgeon may afford the patient the advantages of minimally invasive surgery.

## Figures and Tables

**Table 1:** Reported incidence of marginal ulceration of GJA after gastric bypass.

Author	Incidence of GJA ulceration (%)
Suggs et al. [1]	6.3
Higa et al. [12]	1.4
Gonzalez et al. [2]	0
Luján et al. [13]	3.4
DeMaria et al. [14]	5.1
Kligman et al. [3]	0.6
Schwartz et al. [4]	0.8
MacLean et al. [15]	16
